# Short- and Long-Term Neurobehavioral Effects of Developmental Exposure to Valproic Acid in Zebrafish

**DOI:** 10.3390/ijms25147688

**Published:** 2024-07-13

**Authors:** Marina Ricarte, Niki Tagkalidou, Marina Bellot, Juliette Bedrossiantz, Eva Prats, Cristian Gomez-Canela, Natalia Garcia-Reyero, Demetrio Raldúa

**Affiliations:** 1 Institute for Environmental Assessment and Water Research (IDAEA-CSIC), 08034 Barcelona, Spain; marina.ricarte@idaea.csic.es (M.R.); niki.tagkalidou@idaea.csic.es (N.T.); juliette.bedrossiantz@idaea.csic.es (J.B.); 2 Department of Analytical and Applied Chemistry, School of Engineering, Institut Químic de Sarrià, Universitat Ramon Llull, 08017 Barcelona, Spain; marina.bellot@iqs.url.edu (M.B.); cristian.gomez@iqs.url.edu (C.G.-C.); 3 Research and Development Center (CID-CSIC), 08034 Barcelona, Spain; eva.prats@cid.csic.es; 4 Institute for Genomics, Biocomputing & Biotechnology (IGBB), Mississippi State University, Starkville, MS 39762, USA; natalia@icnanotox.org

**Keywords:** valproic acid, animal model, zebrafish, autism spectrum disorder, social behavior, neurotransmitter

## Abstract

Autism spectrum disorder (ASD) is a neurodevelopmental disorder characterized by impairments in social interaction and communication, anxiety, hyperactivity, and interest restricted to specific subjects. In addition to the genetic factors, multiple environmental factors have been related to the development of ASD. Animal models can serve as crucial tools for understanding the complexity of ASD. In this study, a chemical model of ASD has been developed in zebrafish by exposing embryos to valproic acid (VPA) from 4 to 48 h post-fertilization, rearing them to the adult stage in fish water. For the first time, an integrative approach combining behavioral analysis and neurotransmitters profile has been used for determining the effects of early-life exposure to VPA both in the larval and adult stages. Larvae from VPA-treated embryos showed hyperactivity and decreased visual and vibrational escape responses, as well as an altered neurotransmitters profile, with increased glutamate and decreased acetylcholine and norepinephrine levels. Adults from VPA-treated embryos exhibited impaired social behavior characterized by larger shoal sizes and a decreased interest for their conspecifics. A neurotransmitter analysis revealed a significant decrease in dopamine and GABA levels in the brain. These results support the potential predictive validity of this model for ASD research.

## 1. Introduction

Autism spectrum disorder (ASD) is a neurodevelopmental disorder characterized by impairments in social interaction and communication, anxiety, hyperactivity, and interest restricted to specific subjects [1,2,3]. It is believed that the emergence of autism involves a combination of anatomical brain abnormalities, genetic anomalies, and neurochemical imbalances [2,4,5]. Epidemiological studies emphasize the significance of genetic factors, revealing that autistic disorder ranks among the most genetically influenced neuropsychiatric conditions [6]. While candidate genes implicated in autism primarily involve proteins that regulate neuronal network patterning and the balance between excitatory and inhibitory signaling, ASD is highly polygenic, with no specific gene or locus associated with a large group of patients [6,7,8]. Approximately 20% of patients exhibit genetic mutations affecting known major genes, contributing to the complexity of clinical manifestations and etiology, without a specific biological hallmark identified for this disorder [2]. In addition to the genetic factors, multiple environmental factors have been related to the development of ASD [1,3,6,7].

Animal models in ASD research can serve as crucial tools for understanding the complexity of the disorder, as they allow testing specific hypotheses of its causes and identifying potential therapeutic strategies [9,10]. However, challenges persist in creating models that faithfully represent the complexity of ASD due to its unclear etiology and heterogeneous symptomatology [9]. Replicating ASD symptoms through animal models is crucial for the identification of the neurobiological basis associated with it [2].

Molecular genetic models allow for the simulation of core features of human autism, including the disruption of glutamatergic synaptic transmission, loss of inhibitory GABAergic interneurons, and impairments in synaptic plasticity [11]. However, while mammalian models expressing autism-linked genetic mutations have been developed, their utility is constrained by the associated costs. Thus, simpler models, such as *Danio rerio*, are proposed for initial mechanistic and screening studies [7]. Zebrafish, a widely used model in biomedical research, emerges as an ideal candidate for behavioral screening in ASD research, as its predominant social behavior along with other advantages make it a valuable tool for investigating the neurobiological basis of the disorder [12,13,14].

Environmental factors, such as drug and toxin exposure; viral infections; and immune dysfunctions during pre- and postnatal stages can influence ASD risks [6,7,8]. For instance, prenatal exposure to valproic acid (VPA) has been associated with an increased incidence of autism [7,15]. Because of the association observed in humans between maternal treatment with VPA and ASD, the use of prenatal VPA exposure has been used to build chemical models in different animal models, including zebrafish, to study ASD [9,16,17]. VPA dysregulates key transcription factors; signal transduction pathways; inositol metabolism; and direct modulation of epigenetic regulators, specifically histone deacetylases (HDACs), resulting in more compact DNA-histone packaging and reduced gene transcription [8,9]. Moreover, VPA exposure has been reported to lead to changes in gamma-aminobutyric acid (GABA) levels in the central nervous system [18,19,20].

In this study, an integrative approach combining behavioral analysis and neurotransmitters profile has been used, for the first time, to determine the effects of early-life exposure to VPA both in the larval and adult stages. With this aim, 4 h post fertilization (hpf) zebrafish embryos have been exposed to 48 mM VPA for 48 h, and then, transferred to clean fish water. The effect of early-life exposure to VPA on basal locomotor activity and the escape response evoked by visual and vibrational stimuli were determined at 8 days post-fertilization. At the adult stage (3 months), social behavior (shoaling and social preference tests) was analyzed, and metabolomic changes in the brain were determined.

## 2. Results

### 2.1. Systemic and Developmental Effects in Zebrafish Larvae Exposed to VPA during Early Development

Embryos exposed to VPA exhibited a significant delay in hatching time compared to controls. At 48 hpf, around 40% of the control embryos, but none of those exposed to VPA, had hatched (Supplementary Figure S1A; *z* = −8.33, *p* < 0.0001). At 72 hpf, 95% of the control and 80% of the VPA-exposed embryos had hatched (Supplementary Figure S1B; *z* = −5.75, *p* < 0.0001).

When the toxicity of the VPA treatment was determined in 7 dpf larvae, the VPA-exposed group exhibited a similar cumulative mortality to the control group (Supplementary Figure S1C). However, VPA exposure led to a mild but significant increase in phenotypic abnormalities, such as pericardial and yolk sac edemas (Supplementary Figure S1D; 0.83% and 4.65%, median values for control and VPA, respectively; *z* = −3.52, *p* < 0.0001).

VPA exposure during early development (4–48 hpf) also led to a significant increase in heart rate when larvae reached 8 dpf, compared to the corresponding controls (Supplementary Figure S2; *t* (31) = 3.504, *p* = 0.0014).

### 2.2. Neurobehavioral Effects in Zebrafish Larvae Exposed to VPA during Early Development

**Figure 1:**
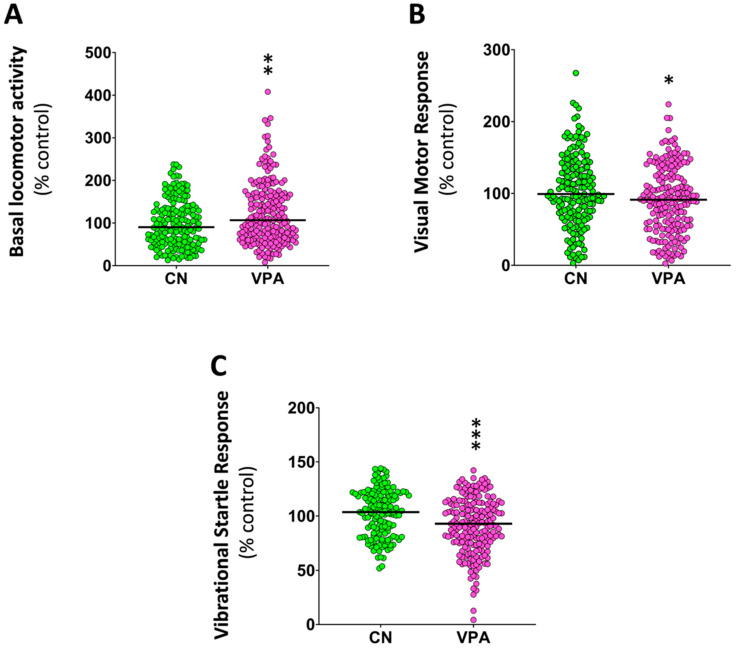
Effects of embryonic exposure to VPA on the behavior of zebrafish larvae. (**A**) Percentage of basal locomotor activity relative to the control; (**B**) percentage of light-off visual motor response relative to the control; (**C**) percentage of vibrational startle response relative to the control. Data reported as a scatter plot with the median (*n* = 160–209). * *p* < 0.05, ** *p* < 0.01, *** *p* < 0.001; Mann–Whitney U test. Data from 3 independent experiments.

For neurobehavioral assessment, 8 dpf zebrafish larvae without any evidence of systemic toxicity were selected. Moreover, no differences in the standard length were found between the control and the VPA-treated groups [*t* (18) = 1.852, *p* = 0.08]. As shown in Figure 1, the exposure of embryos to VPA from 4 to 48 hpf led to significant changes in all three analyzed behaviors. While BLA increased in the treated larvae (*z* = −2.92, *p* = 0.0034), the escape response evoked by a visual and a vibrational stimulus decreased (*z* = −2.35, *p* = 0.0185 for VMR; *z* = −3.86, *p* < 0.0001 for VSR).

**Figure 2:**
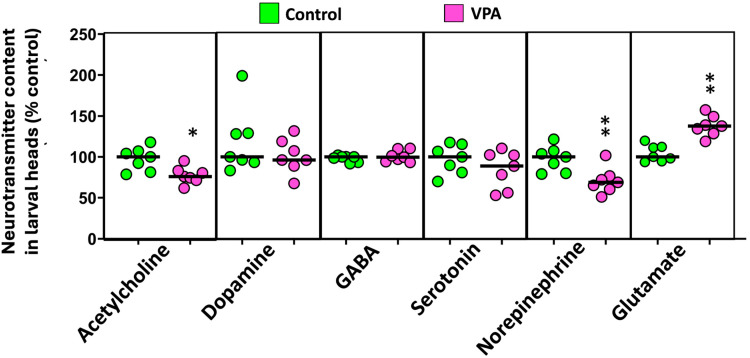
Effects of embryonic exposure to VPA on the neurotransmitter profile of zebrafish larvae. The neurotransmitters analyzed in the larval heads include catecholamines (dopamine, norepinephrine, and epinephrine), serotonin, acetylcholine, GABA, aspartic acid, and glutamate. Data reported as a scatter plot with the median (*n* = 7) * *p* < 0.05, ** *p* < 0.01; Mann–Whitney U test. Data from two independent experiments.

The following step was used to determine if the observed changes in the different behaviors might be related to changes in the neurotransmitter profile of larvae exposed to VPA during early development. As shown in Figure 2 and Supplementary Table S1, larval heads exposed to VPA during early development presented a significant reduction in the levels of acetylcholine (U(*N*_control_ = 7, *N*_VPA_ = 7) = 6.00, *p* = 0.017) and norepinephrine (U(*N*_control_ = 7, *N*_VPA_ = 7) = 4.00, *p* = 0.007), whereas the levels of glutamate increased in this group (U(*N*_control_ = 7, *N*_VPA_ = 7) = 48.00, *p* = 0.001). VPA-exposed larvae also showed a significant decrease in epinephrine levels (U(*N*_control_ = 7, *N*_VPA_ = 7) = 2.00, *p* = 0.002; Supplementary Table S1).

### 2.3. Neurobehavioral Effects in Adult Zebrafish Exposed to VPA during Early Development

The adult zebrafish selected for behavioral analysis had a similar body length (19.97 ± 0.28 mm for the control and 18.95 ± 0.34 for the VPA-treated group), and no differences in the total distance moved in 6 min were found between the VPA-exposed and control fish (Supplementary Figure S2).

**Figure 3:**
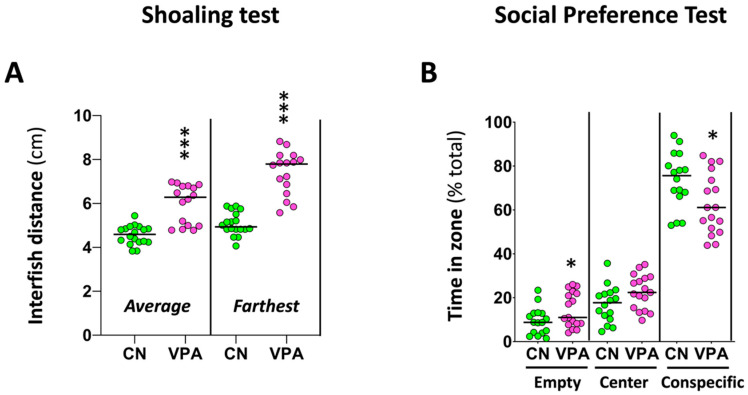
Effect of early-life VPA exposure on social behavior. (**A**) VPA exposure during the early embryonic development increases the average interfish distance and the farthest interfish distance in the shoaling test. (**B**) VPA exposure during early embryonic development decreases the time that fish spend in the zone closest to conspecifics, increasing, however, the time spent in the empty zone in the social preference test (SPT). In the SPT, the time spent in each virtual zone for each fish was normalized to the total time of the assay. Data reported as a scatter plot with the median (*n* = 16–18) * *p* < 0.05, *** *p* < 0.001; *t*-test or Mann–Whitney U test. Data from two independent experiments.

The effects of early-life exposure to VPA on adult social behavior were explored using two experimental paradigms, the shoaling test and the social preference test. The shoaling test (Figure 3A) showed significantly higher average interfish distance (U(*N*_control_ = 18, *N*_VPA_ = 16) = 261.00, *p* = 1.28 × 10^−5^) and farthest interfish distance (*t* (32) = −8.667, *p* = 6.65 × 10^−10^), a behavioral phenotype consistent with social isolation, in the adults exposed to VPA during early development. The social preference test (Figure 3B) showed a significant decrease in both the time spent and distance moved by treated fish in the zone closest to conspecifics (time: *t* (31) = 2.350, *p* = 0.025; distance: *t* (31) = 2.348, *p* = 0.025), and a concomitant increase in the time spent and the distance moved by these fish in the empty virtual zone (time: *t* (31) = −2.112, *p* = 0.043; distance: *t* (31) = −2.125, *p* = 0.042). These results are consistent with the social isolation phenotype suggested by the shoaling test results.

**Figure 4:**
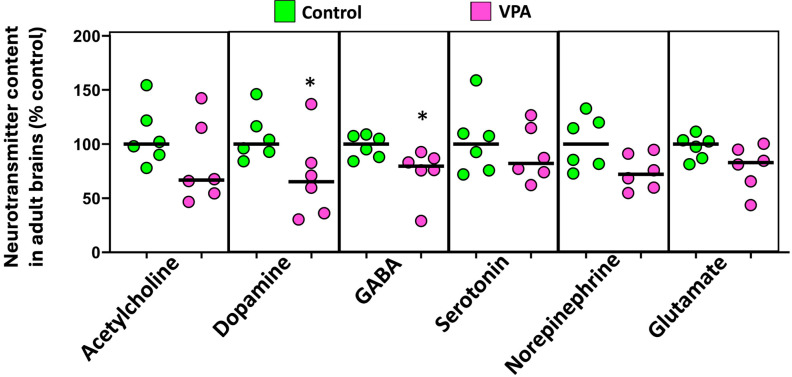
Effects of embryonic exposure to VPA on a neurotransmitter profile in the brain of an adult zebrafish. The neurotransmitters analyzed include catecholamines (dopamine and norepinephrine), serotonin, acetylcholine, GABA, and glutamate. Data reported as a scatter plot with the median (*n* = 6) * *p* < 0.05; Mann–Whitney U test. Data from two independent experiments.

As shown in Figure 4, when the levels of different neurotransmitters in the brain were analyzed, a significant decrease in the levels of GABA (U(*N*_control_ = 6, *N*_VPA_ = 6) = 3.00, *p* = 0.015) and dopamine (U(*N*_control_ = 6, *N*_VPA_ = 6) = 5.00, *p =* 0.041) was found in VPA-exposed fish compared to controls. A trend to a reduction in glutamate levels was also found in the brain of the exposed fish (U(*N*_control_ = 6, *N*_VPA_ = 6) = 6.00, *p* = 0.065).

## 3. Discussion

VPA is a medication widely used to treat epilepsy, bipolar disorder, and migraines [21]. Despite its therapeutic effect, exposure to VPA during the first three months of pregnancy can result in a rare condition known as fetal valproate syndrome (FVS) [21]. Children with FVS also have an increased risk of ASD [22,23]. In fact, VPA is commonly used to build chemical models of ASD in different animal species, including zebrafish [12,24]. In this study, an integrative approach combining behavioral analysis and neurotransmitter profiles has been used, for the first time, to determine the effects of early-life exposure to VPA both in the larval and adult stages. First of all, we have found that the exposure of 4 hpf zebrafish embryos to 48 μM VPA for 48 h did not result in mortality when this endpoint was evaluated at 7 dpf. Consistently, no mortality in zebrafish embryos has been reported by other authors using the same [12] or similar exposure conditions [25] to VPA. However, the effect of VPA exposure on zebrafish embryo mortality is, anyway, controversial. Whereas in one report, embryos exposed to 40 μM VPA from 8 to 120 hpf showed a 96% mortality at 7 dpf [26], in another report, the exposure of 8 hpf zebrafish embryos for 100 h to 1500 μM VPA did not result in mortality [16].

In our study, we have found that exposure of zebrafish embryos during the first 48 h of development to VPA led to a mild (4.65%) but significant increase in phenotypic abnormalities [21]. In contrast, no phenotypic effects have been reported in exposed embryos by other authors using similar exposure conditions [12,25]. While morphological alterations were also not reported after exposure of embryos to 75 μM VPA from 4 to 120 hpf [17] or to 50 μM VPA from 8 to 108 hpf [16], exposure to 30 μM VPA from 8 to 120 hpf showed 30.5% malformations.

The observed variability in mortality and malformation rate among these studies based on waterborne exposure to VPA could be related to the differences in the final pH of the experimental solutions. VPA is a weak acid with a pK_a_ of 4.86, and depending on the pH of the experimental solution, the chemical will be ionized (low bioavailability) or non-ionized (high bioavailability). The percentage of non-ionized VPA molecules is higher at low pH (39% of VPA is in its non-ionized form at pH 5.0) than at the physiological pH 6.3 (3.1% non-ionized) [27]. Therefore, VPA uptake should be significantly higher in studies using experimental solutions with low pH than in those using experimental solutions with a final pH above 7. In fact, when we performed the first experiments with VPA, using our standard fish water to prepare the experimental solution of VPA (pH 6.5), we found a huge mortality of exposed embryos. At pH 6.5, about 1.96% of the VPA is expected to be in the form of valproate (non-ionized form) and, therefore, bioavailable. The pH of the fish water was then raised to 7.5 in both fish water and experimental solutions by adding bicarbonate, and at this pH, the non-ionized fraction should be only 0.23%. When this water was used to prepare the experimental solutions, we found no mortality and a very limited percentage of malformations. In addition to the potential differences in VPA bioavailability, the sensitivity of the zebrafish strain and batch could have also played a role, as some malformations were observed on the controls too.

The hyperactivity found in VPA-exposed larvae in this study aligns with the results obtained by most of the previous studies using this chemical [12,16,26,28] and may be related to the dysregulation of dopaminergic and glutamatergic neurotransmission in certain brain regions, as suggested for ADHD (attention-deficit hyperactivity disorder), a disorder related to and co-occurring with ASD [29,30,31,32]. However, no effects on BLA have been reported in *shank3b*^−/−^ zebrafish larvae, a result suggesting that hyperactivity is a VPA effect unrelated to ASD [14].

In this study, we have also found a decrease in the light-off VMR in larvae exposed to VPA during early development, a result consistent with that reported by Bailey et al. [33] in 6 dpf larvae exposed to 30–50 μM VPA from 4 to 120 hpf, but not with the significant increase reported by Joseph et al. [26] in 5 and 7 dpf larvae exposed from 8 to 108 hpf to 5–10 μM VPA and by Baronio et al. [34] in 5 dpf larvae exposed to 25 μM VPA from 10 to 24 hpf. A similar decrease in light-off VMR was also reported in *shank3b*^−/−^ zebrafish larvae, suggesting that this effect of VPA could be related to ASD. The decrease in the VMR could be related to the excitatory–inhibitory imbalance in the visual cortex suggested to happen in ASD patients [34]. We also observed a significant decrease in the vibrational-evoked startle response in the larvae exposed to VPA during early embryonic development. Interestingly, Gupta et al. [25], in a seminal article on neuroanatomical mapping of the zebrafish brain, found that 6 dpf zebrafish larvae exposed to 50 μM VPA during early development presented a loss of glutamatergic signal in part of the statoacoustic ganglion, leading to a decreased acoustic startle responsiveness. Glutamate dysregulation is thought to be implicated in the changes in startle response through the NMDA and mGlu5 receptors [34,35,36,37].

It is difficult to explain the behavioral changes found in the VPA-exposed larvae based on the observed changes in the neurotransmitter profiles found in their heads. For instance, hyperactivity has been linked to the increase in the dopamine, acetylcholine [38,39], and/or norepinephrine [40,41] levels in the zebrafish larvae. However, the levels of acetylcholine and norepinephrine in the heads of the exposed larvae decreased, and no changes were found in dopamine levels. The observed hyperactivity is also not explained by the increased glutamate levels in the head of VPA-exposed larvae, as glutamate has been reported to reduce the motor activity of zebrafish larvae [42]. In our opinion, the difficulty of correlating behavioral changes with changes in the neurotransmitter profile observed in the larval heads has two components. First, the UHPLC-MS/MS analysis used in this study provides information on the entire set of neurotransmitters present in the larval head, and not only those released in the synaptic spaces, which are the ones directly involved in behavior. This is a clear limitation of the zebrafish model, in which, due to its small size, it is not possible to use microdialysis [43] to selectively determine neurotransmitters in the synaptic clefts of specific CNS nuclei, as for example is conducted in rats. The second limitation of this methodology is that the analysis is performed on the entire head, not discrete nuclei. It is often reported that a chemical increases the levels of a neurotransmitter in some nuclei and decreases them in others. Therefore, even if the total pool of these neurotransmitters does not change in the context of the whole brain, small differences in the neurotransmitter released at specific nuclei of the brain could explain the observed behavioral changes.

The decrease in the social behavior found in this study in adult zebrafish (90 dpf) exposed to VPA during early embryonic development is consistent with previous studies reporting increased size of the shoals [33] and decreased preference for being near conspecifics [12] in VPA-treated zebrafish. Similar disorders of social behavior have been described in genetic models of ASD constructed in zebrafish [13,14]. Interestingly, the decrease found in the dopamine levels in the brain of the VPA-treated fish has also been reported in different mammalian models [44,45]. Since the dopaminergic system is involved in social behavior, the observed decrease in dopamine levels could be behind the impaired social behavior found in ASD [44,46,47]. Moreover, the significant decrease in GABA levels found in the brain of adult fish exposed to VPA during early development is consistent with the decrease in this neurotransmitter reported in ASD patients [48,49].

In summary, the neurobehavioral short- and long-term effects of developmental exposure to valproic acid in zebrafish have been analyzed, with special emphasis on determining the relationship between behavioral and neurochemical changes. Our results suggest the importance of the pH of the experimental solution on the bioavailability of the VPA and, therefore, on the severity of the observed effects. Moreover, our results show that the developed ASD model exhibited an impaired social behavior, characterized by increases in the average inter-fish distance and in the shoaling test and by decreases in the time spent and distance moved in the closest zone to conspecifics and in the social preference test. The impairment of social behavior combined with the significant decrease found in the brain levels of dopamine and GABA in adult zebrafish treated with VPA during early development strongly suggests that this model could be of great interest for both the study of FVS and ASD. However, additional efforts are needed to determine the predictive validity of this model in ASD research.

## 4. Materials and Methods

### 4.1. Zebrafish Housing and Husbandry

Adult zebrafish were supplied by Pisciber BSF (Terrassa, Barcelona, Spain) and maintained at the Research and Development Center of the Spanish Research Council (CID-CSIC) facilities in fish water [FW: reverse-osmosis purified water containing 90 μg/mL of Instant Ocean^®^ (Aquarium Systems, Sarrebourg, France), 0.58 mM CaSO_4_·2H_2_O, pH 6.5] at 28 ± 1 °C. A 12:12 light–dark photoperiod was used. The fish were fed twice a day with flake food (TetraMin, Tetra, Germany).

For breeding, a series of polycarbonate crossing tanks were prepared with a total of five adult zebrafish in each one, with the ratio of female:male being 3:2. After a treatment with 0.1% methylene blue, the embryos were maintained in crystallizing dishes with fish water inside the incubator (at 28 °C, 12:12 light–dark photoperiod). At 4 hpf, the embryos at the sphere and dome stages [50] were selected and transferred to 6-well cell culture plates (20 embryos/well) with the exposure medium.

All procedures were approved by the Institutional Animal Care and Use Committees at the CID-CSIC and conducted in accordance with the institutional guidelines under a license from the local government (agreement number 11336).

### 4.2. Experimental Procedure

Sodium valproate (VPA; CAS: 1069-66-5) was purchased from Sigma-Aldrich (St Louis, MO, USA). For treatment, an exposure protocol similar to that described by Zimmermann et al. (2015) was used [12]. Basically, the day of the experiment, a fresh 48 µM VPA solution was prepared in FW, and then, pH was adjusted to 7.5 with a 1 M NaHCO_3_ solution. At 4 hpf, embryos at sphere-dome stages were exposed to the VPA solution (treated group) or to FW (also adjusted to pH 7.5, control group) for 48 h without medium renewal. Zimmermann et al. (2015) reported that this concentration of VPA is stable in fish water for at least 48 h [12]. At 52 hpf, the embryos were removed from the experimental solutions, washed, and transferred to pH 7.5 FW (28 °C and 12L:12D photoperiod) until 8 dpf, when the behavioral effects of early exposure to VPA were initially tested. Afterwards, the larvae were placed in a 2 L tank with fish water and fed twice a day. After three months, when the zebrafish reached the adult state, social behavior was determined. Finally, the fish were euthanized by inducing a hypothermic shock in ice-chilled water (2–4 °C), and the brains were immediately dissected and individually stored at −80 °C for neurotransmitter and transcriptional analysis.

### 4.3. Phenotypic Analysis

Live embryos and larvae were examined with a Nikon SMZ 1500 stereomicroscope (Nikon, Champigny sur Marne, France) every 24 h to observe the phenotype, recording the lethality, malformations, and hatching time in both the control and VPA-exposed groups. The standard length of the animals from both experimental groups was determined at 8, 30, 60, and 90 dpf from pictures taken with a GigE camera mounted on the stereomicroscope using the GIMP software (version 2.10.32).

### 4.4. Neurobehavioral Assay in Larvae

Behavioral assays including basal locomotor activity (BLA), light-off visual motor response (VMR), and vibrational startle response (VSR) assay were conducted and analyzed in a DanioVision platform driven by EthoVision XT 13 software (Noldus, Wageningen, The Netherlands). The behavioral assays in the larvae were conducted as previously described [51,52].

At day 7, larvae with no signs of systemic toxicity were placed in 48-well microplates, with 1 larva per well in 1 mL FW. For the behavioral analysis, the larvae were first acclimated to the new environment inside the observation chamber for 10 min in darkness, after which the plate received one vibrational stimulus by means of a solenoid (tapping stimulus). The distance (cm) moved in response to the tapping routine corresponds to the vibrational startle response (VSR). After delivering the tapping, the larvae were maintained in the dark without any stimuli for 15 min. The last 10 min in the dark were used to calculate their basal locomotor activity (BLA), corresponding to the total distance moved during that time. The routine continued by switching a light on for 10 min and then turning it off for 15 more minutes. The light-off visual motor response (VMR) was calculated as the distance moved in the first two minutes of darkness minus the distance moved in the last two minutes of light. After the behavioral battery, the larvae were euthanized by inducing a hypothermic shock in ice-chilled water (2–4 °C) and then their heads were cut. Eight pools of fifteen heads were prepared for each experimental condition and stored at −80 °C.

### 4.5. Neurobehavioral Assays in Adults

All tests were conducted in an isolated behavioral room at 27–28 °C. To assess the effects on social behavior, a shoaling and social preference test (SPT) was performed following the protocol described by Bedrossiantz et al. [53]. Ethovision XT 13.0 (Noldus, Wageningen, The Netherlands) was used for the video-tracking analysis. After these tests, the fish were euthanized by inducing hypothermic shock in ice-chilled water (2–4 °C), and the brain of each individual was dissected and transferred into an Eppendorf tube. Eight brains of each concentration were used for the analysis. During all of the processes, the samples were maintained on ice. When all the samples were prepared, they were kept in a freezer at −80 °C until the day of the extraction.

### 4.6. Neurochemical Analysis by UHPLC-MS/MS

For the neurochemical extraction of larval heads and adult brains and UHPLC-MS/MS, a protocol similar to that described by Ricarte et al. [51] was used. Basically, samples were homogenized by means of a bead mill (TissueLyser LT, Quiagen, Hilden, Germany) and centrifuged. The supernatant was filtered, using 0.22 μm nylon filter, into chromatographic vials that were kept at −20 °C until the analysis. To extract and conduct the analysis of neurotransmitters, acetonitrile (ACN), HPLC-MS grade, was supplied from VWR chemicals Prolabo (Leuven, Belgium); formic acid (FA) from Fisher Scientific (Loughborough, UK); and ammonium formate from Sigma-Aldrich (St. Louis, MO, USA). Ultra-pure water was obtained through the Millipore Milli-Q purification system (Millipore, Bedford, MA, USA).

The neurochemical content of the extract was determined by UHPLC–MS/MS, using conditions described elsewhere [54,55]. A BEH Amide column was used for separation and elution, and the detection was performed in MRM mode with ESI+, ensuring specificity during detection and quantification. For the calibration curve, pure reference standards were used: serotonin hydrochloride (5-HT), dopamine hydrochloride (DA), γ-aminobutyric acid (GABA), epinephrine (Epi), and acetylcholine (ACh) were supplied by Sigma-Aldrich (St. Louis, MO, USA). Glutamic acid (Glu) was supplied by BLD Pharmatech (Shanghai, China), and norepinephrine (NE) was obtained from Tocris Bioscience (Ellisville, MO, USA). A mixture of isotopically labelled standards was used as an internal standard. 5-HIAA-d5, 5-HTP-d4, 5HT-d4, NE-d6, DA-1,1,2,2-d4, and 3-MT-d4 were purchased from Toronto Research Chemicals (TRC, Toronto, ON, Canada).

### 4.7. Statistical Analysis

The data were analyzed with IBM SPSS v29 (Statistical Package 2010, Chicago, IL, USA) and GraphPad Prism 9 for Windows (GraphPad software Inc., La Jolla, CA, USA) and plotted with GraphPad Prism 9 for Windows (GraphPad software Inc., La Jolla, CA, USA).

In order to determine if the samples followed a normal distribution, a Shapiro–Wilk test was used. For normally distributed groups, an Unpaired *t*-test was used for determining statistical significance and one-way ANOVA followed by Dunnett’s as a multiple comparison test. When parametric assumptions could not be made, statistical significance was determined by a Mann–Whitney U test and a Kruskal–Wallis test followed by Dunn–Bonferroni’s test to see if there were any differences between more than two groups. Significance was set at *p* < 0.05.

## Data Availability

The data supporting the findings of this study are available within the manuscript and its Supplementary Material file or will be made available from the corresponding author upon request.

## Supplementary Materials

The following supporting information can be downloaded at https://www.mdpi.com/article/10.3390/ijms25147688/s1.
