# Supplementary material for: Short- and Long-Term Neurobehavioral Effects of Developmental Exposure to Valproic Acid in Zebrafish
Source: Int J Mol Sci. 2024 Jul 13;25(14):7688. doi: 10.3390/ijms25147688 (PMC11277053; doi:10.3390/ijms25147688)
Supplement: Supplementary file 1 [file ijms-25-07688-s001.zip › ijms-3091666-supplementary.pdf]

Supplementary Material

Short- and long-term neurobehavioral effects of the developmental exposure to valproic acid in zebrafish

Marina Ricarte, Niki Tagkalidou, Marina Bellot, Juliette Bedrossiantz, Eva Prats, Cristian Gomez-Canela, Natalia Garcia-Reyero, Demetrio Raldúa

Supplementary Figures

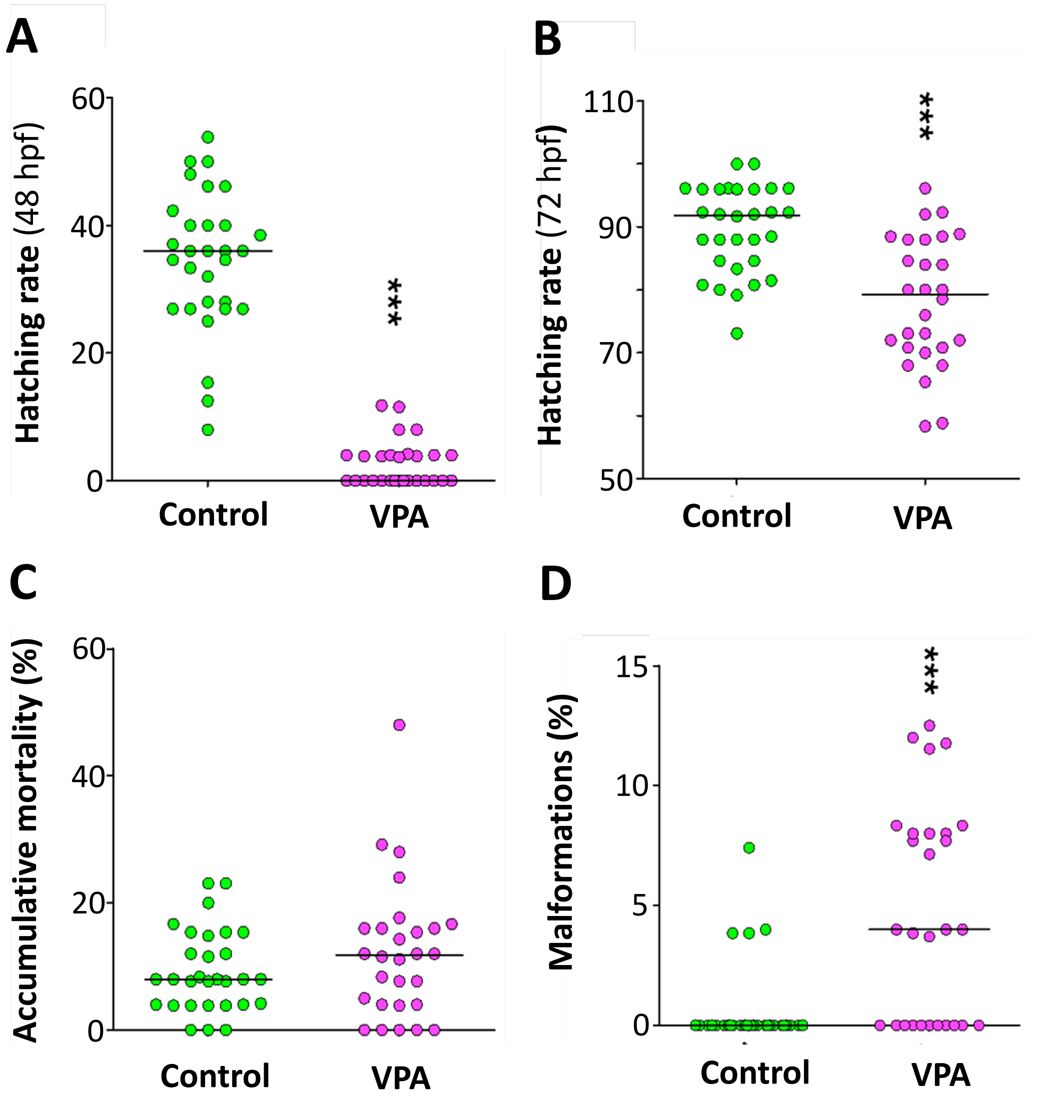

Figure S1. Systemic and developmental effects in zebrafish larvae. (A) percentage of hatched embryos at 48 hpf (Mann-Whitney U test); (B) percentage of hatched embryos at 72 hpf (unpaired t-test); (C) percentage of cumulative mortality at 8dpf (Mann-Whitney U test); (D) percentage of embryos with malformations at 7 dpf (Mann-Whitney U test). Data reported as scatter plot with median (n = 28-30). *p < 0.05, **p < 0.01, ***p < 0.001.

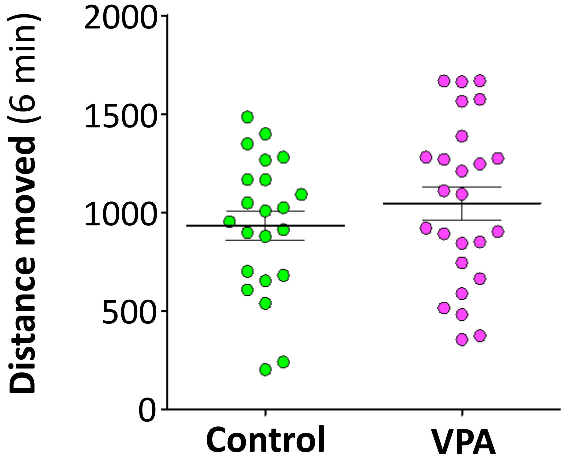

Figure S2. Total distance moved in 6 minutes (cm). Data reported as scatter plot with the mean (n = 22-25, Unpaired *t-test*). $^*p < 0.05$, $^{**}p < 0.01$, $^{***}p < 0.001$.

Supplementary Tables

Supplementary Table S1: Statistical results from Mann-Whitney U test in neurotransmitter levels of larvae heads.

Metabolite	Group	N	*U*	*p*
**Acetylcholine**	CN	7	6	0.017*
	VPA	7		
**Dopamine**	CN	7	19	0.535
	VPA	7		
**GABA**	CN	7	28	0.710
	VPA	7		
**Epinephrine**	CN	7	2	0.002**
	VPA	7		
**Serotonin**	CN	7	16	0.318
	VPA	7		
**Norepinephrine**	CN	7	4	0.007**
	VPA	7		
**Glutamate**	CN	7	48	0.001**
	VPA	7		

*p < 0.05, **p < 0.01
